# Bridging psychiatric and educational metacognition: a perspective on insight and learning

**DOI:** 10.3389/fpsyt.2026.1831296

**Published:** 2026-08-26

**Authors:** Krisztina Szalisznyó, David N. Silverstein

**Affiliations:** 1 Department of Psychiatry, Uppsala University Hospital, Uppsala, Sweden; 2 Department of Medical Sciences, Clinical Psychiatry, Uppsala University, Uppsala, Sweden; 3 Theoretical Neuroscience and Complex Systems Research Group, HUN-REN Wigner Research Centre for Physics, Budapest, Hungary; 4 NeuroAI Institute, Boston, MA, United States

**Keywords:** metacognition, metacognitive training, disease insight, illness management and recovery, insight paradox

## Abstract

This perspective considers some commonalities across metacognitive disturbances in psychiatric disease insight and teaching metacognitive awareness in learning environments. Psychiatry and educational disciplines developed in parallel, with limited interaction. We argue that there is a relatively unexplored overlap between metacognitive processes during learning skill development in educational settings and addressing pathological, impaired metacognition. We claim that these two challenges can inform each other, leading to a better understanding of the metacognitive brain. The question is particularly interesting from a psychiatric point of view, as altered metacognitive abilities during learning processes and disease insight disturbances crucially influence therapeutic outcomes.

## 1 Introduction

Metacognition is cognition of cognition, an awareness of one’s own thought processes and includes both metacognitive knowledge and metacognitive regulation (1–4). These capacities help to regulate cognition and maximize the potential to think more efficiently. Metacognition and insight are both domain-specific, multidimensional and closely connected. Underlying metacognitive abilities are the mechanisms that enable aspects of insight and particular metacognitive deficits result in specific deficiencies of insight.

Daniel Kahneman describes two modes of thought during decision making, one fast, automatic, intuitive and possibly emotional (System-1) and the other slow, effortful, deliberative and analytical (System-2). These systems often can cooperate or sometimes conflict with each other (5). Metacognitive feelings (6) can help decide which system to recruit, with more explicit metacognition utilized in System-2.

Epistemology from the Greek “epistémē” is the branch of philosophy concerned with knowledge and regulation of cognition. Epistemologists study the nature, origin and scope of knowledge, epistemic justification and the rationality of belief (7). Epistemic injustice in psychiatry (8) occurs when a person’s knowledge and description of their own mental states and perceptions are unfairly dismissed or misunderstood.

Insight is a key aspect of well-being and encompasses self-knowledge of internal emotions, thoughts, beliefs and the sense of self (9). Disease insight includes metacognitive awareness of illness, individual symptoms and performance. Patient insight into their psychiatric disorders, such as psychosis and schizophrenia, obsessive compulsive disorder (OCD), anorexia nervosa and personality disorders such as pathological narcissism has major implications on clinical outcomes. Anosognosia, the lack of insight of disease symptoms and deficits, raises practical, ethical and legal challenges in medicine, particularly in psychiatry as it can interfere with treatment (10). There is evidence of an overlap and differences between cognitive and clinical insight. Cognitive insight refers to abilities to understand and reflect upon one’s own mental states and processes (11). Clinical insight includes the ability to relabel symptoms as pathological. More severe clinical symptoms tend to correlate with reduced disease insight, although lower mood can correlate with better insight. One possible reason for this exception is that patients may reflect more on the consequences of the disorder (12). Higher cognitive ability, both general intelligence (IQ) and specific executive functions and memory are associated with better insight (12). When analyzing disease insight, one has to distinguish between local and global processes, as patients can have different calibration accuracy at local and global levels (13).

Mentalization is the ability to understand ones own and others’ thoughts, feelings and intentions (14). Alterations in mentalization can be a trans-diagnostic factor, for example in autism and schizophrenia spectrum disorders, highlighting the potential for trans-diagnostic interventions (15). Mental state attribution or Theory of Mind (ToM) engages specialized social brain areas and is considered to be fundamental for social interactions (16). ToM capabilities enable one to infer and predict the intentions and affective states of others (16).

While broadly, metacognitive training in education (MT-ED) and metacognitive training for disease insight (MT-DI) have different purposes, this perspective article considers some overlaps and how these approaches may inform each other. Knowledge transfer between these disciplines can contribute to mapping domain-general and domain-specific cognitive processes, while increasing the efficacy of personalized interventions.

## 2 Improving patient disease insight

A deeper understanding of illness insight can contribute to designing a psycho-education system for patients with new treatment strategies, based on neuropathology and disease trajectories (17). Metacognitive training (MCT) has been found to reduce overall symptom severity, including positive and disorganized symptoms in schizophrenia patient groups. MCT can improve specific aspects of negative symptoms in schizophrenia as well (18). Metacognitive Reflection and Insight Therapy (MERIT) is based on the hypothesis that increased reflective capacity with broader metacognitive processing for identifying and integrating experiences are central mechanisms for self-directed recovery (19). MERIT uses approaches which explicitly address self-experience in psychosis (20, 21). Targeting mentalization abilities may improve insight deficits in schizophrenia (22) and therapeutic alliance (23). Schizophrenia patients with spontaneous mentalizing deficits may benefit from attention and imitation–inhibition training, which can facilitate perspective-taking abilities (24). Also, enhancing mental state vocabulary may improve mentalization performance (24, 25). The Illness Management and Recovery program (IMR) was developed to teach illness self-management strategies to patients with schizophrenia and other severe mental illnesses (26). Strong emphases are given on how to cope with symptoms, on preventing relapses and on how to improve knowledge of the mental illnesses (26). Often there is a difference between the doctor’s and patient’s narratives on the illness and the cause of symptoms, which can have severe consequences that may lead to compulsory psychiatric treatment (10).

Metacognitive therapies seek to strengthen first-person authority, giving patients a better understanding of experiences to help validate their own perspectives and respectfully address delusional content. This can also reduce the potential for epistemic injustices, considering that psychiatric patients are more vulnerable to this than those with physical disorders only (27). With increased disease insight, other effects can manifest, including self-stigma, reduced self-esteem, negative self-evaluation, increased awareness of reasoning errors and depression. This concept, the insight *paradox*, highlights the possible negative outcomes of improved insight, for example in individuals with schizophrenia (17). Further, metacognitive training is not useful when it undermines first-person authority and creates feelings of epistemic injustice (8, 27). Insight promotion interventions should consider the possible detrimental effects (28). In OCD, the tendency to think too much about one’s thoughts could potentially lead to persistent, excessive reflection and overreactions to thoughts (29). Patients who suffered from severe brain damage (e.g. stroke) in the metacognitive brain circuits might develop anosognosia. With severe damage, metacognitive therapies may not be appropriate and effective (30). In neurodegenerative disorders, anosognosia usually correlates with medial temporal and prefrontal atrophy. With disease progression, both metacognitive monitoring accuracy and metacognitive regulation deteriorates (31, 32). Therefore, insight promotion interventions should address the trajectory of insight changes as well (28). Goal-based cognitive rehabilitation and scaffolding may be more appropriate than metacognitive training in cases of dementia with memory loss and reduced insight (33, 34).

Different methods exist to assess insight, such as the Beck Cognitive Insight Scale (BCIS), which measures self-reflectiveness and self-certainty (35). Considering that insight is a multidimensional capacity, no single instrument captures functional capabilities across clinical populations. The best approach is to use a specific tool, depending on the construct being assessed, target population, cognitive capacity, illness stage, assessment purpose and time burden (36). Another important trans-diagnostic contribution is to consider metacognition as process-level mechanisms (such as error detection, confidence calibration and belief updating), which may have comorbidity or heterogeneity across diagnoses (37). Recent developments in digitalization can move metacognitive assessments from static clinical measurements towards teachable, more flexible and personalized approaches (38).

## 3 Metacognitive skill development in educational settings

In classroom settings, introducing learning tasks with metacognition early in curricula to develop strategies for self-awareness, self-monitoring and control of internal learning processes can have significant impacts. According to constructivism learning theory, knowledge is constructed through experiences and social interactions, rather than just passively by direct instruction. Jean Piaget concluded that learning was a product of an individual mind, integrating experiences achieved through observation and experimentation (39). Lev Vygotsky argued that learning is socially mediated such as through interactions with a teacher or informed peers. Vygotsky regarded the teacher as co-explorer who encourages learners to question, challenge and formulate their own ideas, opinions and conclusions (40, 41). When designing learning programs using a constructivist model, teachers focus on ensuring the learning is active and collaborative, while including elements of problem solving. The students determine their own learning needs, set goals and strategies to achieve these goals and then monitor, control and evaluate their learning. Monitoring implies that learners develop metacognitive awareness of what they know, controlling implies they adapt learning to their needs (42).

One approach based on constructivist pedagogy is problem-based learning (PBL), which can help prepare students effectively for future learning (42). It was developed in the 1960s for medical education to improve clinical reasoning, team skills and to enhance metacognition (43). PBL has been applied in other fields such as law and engineering as well. Standard PBL develops metacognitive skills implicitly, but some variants such as PBL integrated with Reading, Questioning and Answering (PBLRQA) (44) also add some explicit components. When metacognitive training in education is explicit, students are not just taught how to reflect, but also how reflection works as a learning strategy and how to change behaviors on what is observed (45) as well as to plan, monitor and evaluate (46).

Self-regulated learning (SRL) emerged in the 1980s, has similarities to the self-directed learning component of PBL (42) and has several model variants that can contain cognitive, metacognitive, behavioral, motivational and affective aspects of learning (47). Considering two variants, Zimmerman’s SRL (48) focuses on subprocesses for forethought (task analysis, self-motivation), performance (self-control, self-observation) and self-reflection (self-judgment, self-reaction). Metacognitive monitoring is part of self-observation and calibration is a reflection on the accuracy of self-judgment (49). Efklides’ Metacognitive and Affective model of SRL (MASRL) adds emphasis on motivation, affect and volition (6). Efklides considers both top-down (Person level) and bottom-up (Task x Person level) regulation. Metacognition is not considered emotionally neutral and metacognitive experiences both before and during tasks, such as difficulty, confidence, anxiety and satisfaction can alter task progression and outcomes. While SRL is at the individual level, recent extensions consider socially shared regulation among a group on shared tasks and co-regulation, which includes the constraints of others (50). SRL processes have some commonality with clinical settings. For example, Zimmerman’s model considers a regulatory cycle and Efklides’ model addresses affect. In particular, SRL overlaps with illness self-management.

## 4 Possible neurobiological overlaps

Metacognition is a multidimensional capacity and is under segregated neural control (51–53). Structural and dynamical neural correlates of metacognition include several interactive networks and multiple hierarchically organized domains (54). Both domain-specific and domain-general metacognitive processes co-exist in the brain and they rely on partially shared circuitry (37, 55). The neural developmental trajectory of metacognitive abilities can help to identify crucial periods during training, but can also inform neuropathology (56). Without claiming completeness, we highlight some specific metacognitive subdomains with corresponding neurobiological substrates and insight alterations across metacognition in educational training and disease insight. The highlighted subdomains are reflection, regulation, monitoring, confidence pro-cessing, calibration, perspective-shifting and scaffolding.

Self-reflective processing, which insight is most related to, has strong recruitment in the medial prefrontal cortex (mPFC), a subregion of the prefrontal cortex (PFC) and the default mode network (DMN) (57). The DMN is essential for self-referential, autobiographical and internally oriented cognition (58). Cortical thickness in the left superior temporal gyrus (part of the DMN) was better preserved while disease insight improved in schizophrenia patients, who participated in an IMR group as compared to controls (59). Metacognitive brain regions in educational settings include the banks of the superior temporal sulcus, part of the DMN (60). Higher connectivity between the anterior DMN and salience network, as well as between the posterior DMN and salience network have facilitated metacognitive accuracy in traumatic brain injury patients (61). DMN hypometabolism has shown to be linked with anosognosia in Alzheimer’s Disease (AD) (62). Functional abnormalities in DMN has been implicated with insight disturbances (63). The precuneus is a major DMN hub, supporting a link between metacognition and self-referential neural systems in educational settings (60, 64). The precuneus was implicated in metamemory, but also found to be modulated by global self-belief processing (37, 55).

Activity in brain areas including the temporoparietal junction (TPJ), precuneus, dorsomedial PFC (dmPFC), anterior cingulate cortex (ACC) and anterior insula (aI) were identified in the metacognitive representation of self attention, but also in conditions when attention was directed towards others. There is a common overlapping cortical circuit for metacognitive attention, although self and other processing recruits distinct circuits as well (65). Specialized neural circuitry, which is included in the ToM are the right TPJ, bilateral anterior mPFC, right precuneus and these networks overlap with the DMN (16, 66).

The frontoparietal control network (FPCN) includes the dorsolateral PFC (dlPFC), frontal cortex, aI, dorsal ACC (dACC), frontopolar cortex, inferior parietal cortex and contributes to metacognitive regulation. dACC processes task sensitive but generalizable metacognitive-control signals (67). Education-related knowledge and regulation was associated with the rostral ACC, the caudal middle frontal cortex and the precuneus (60). The likely neural substrates of affective regulation include the rostral and subgenual ACC, ventromedial PFC (vmPFC), orbitofrontal cortex, aI, amygdala and lateral PFC (lPFC). In disease insight, the same FPCN is involved, but it also requires coordination with medial self-referential and affective systems, where the later might be especially important because illness-related information may threaten identity and autonomy. The insula integrates internal bodily, cognitive and emotional information with salience and contributes to introspection. The iA signals multimodal representations of the internal state. Insight also depends on assigning personal significance to errors, symptoms and uncertainty and the lack of insight may involve affectively biased interpretation or avoidance, rather than only detecting cognitive errors. A high-resolution MRI structural study supported the disease insight relevance of mostly right sided insular/salience dysfunction in schizophrenia and psychosis (68).

Monitoring is an ongoing detection of errors, conflict and discrepancies between expected and actual outcomes. ACC plays a role in this, with the dACC monitoring regulation across different domains (67). While activity in the left inferior frontal gyrus increases in children during metacognitive monitoring in educational settings (69), left inferior frontal and supramarginal gyrus deficits were found in schizophrenia patients with impaired insight (70). The FPCN is associated with performance monitoring. Patients with traumatic brain injury were less able to identify their own errors and showed reduced functional connectivity within this network (71). Additionally, event-related potential alterations evoked by erroneous responses, support the idea that anosognosia may arise from dysfunction in neural error-monitoring systems in AD (72).

Confidence processing is the ability to evaluate choices and certainty. The mPFC, in particular the perigenual anterior cigulate cortex, tracks expected performance, a correlate of decision confidence. One study found that when confidence-based behavioral control was successful, top-down negative modulation of the dmPFC via the vmPFC occurred (73). In vmPFC, local confidence signals are modulated also by a global metacognitive evaluation of self-belief processing (37). Further, both the striatum and the insula contribute to perceived confidence processing. Calibration of confidence, certainty and belief relies on the anterior PFC and dlPFC (74). Structural variations in the anterior PFC and disruption of lPFC have both been associated with changes in metacognitive accuracy (75). Students with more accurate metacognitive calibration and efficacy showed higher lPFC recruitment when detecting errors than their peers when evaluating error-containing models in educational settings (49).

Perspective-shifting and perspective-taking are the abilities to move between different self and other viewpoints to obtain a more detached or decentered interpretation. The principal network includes TPJ, mPFC, lPFC, precuneus, superior temporal sulcus and temporal poles (76). In educational settings, perspective-shifting occurs when a learner compares their reasoning with an expert explanation. In disease insight, perspective shifting is often crucial.

Metacognitive scaffolding is a systematically organized external support that prompts planning, monitoring and regulation. Scaffolding recruits multiple brain systems, which includes dACC, aI for conflict and discrepancy detection, lPFC for strategic control, hippocampal systems for retaining procedures, mPFC and TPJ for self-reflection and another person's perspective. Educational metacognitive scaffolding in children increased an electrophysiological index of conflict processing, which is associated with activation arising in the ACC and these neural changes predicted intelligence gains (77). Neural studies combining metacognitive scaffolding with attention training support that scaffolding strengthens conflict monitoring and regulation (31, 77).

## 5 Blended insight: integrating MT-ED for education and MT-DI for disease insight

With MT-ED, metacognition is applied while learning a particular subject such as physics, whereas with MT-DI, the focus is on understanding and coping with a personal illness. However, they share core mechanisms:

• reflection of cognition, behavior and affect
• metacognitive knowledge about self, tasks and strategies
• metacognitive regulation, strategy selection and control
• monitoring and evaluation
• calibration of confidence, certainty, and appraisal
• affective regulation of difficulty, motivation
• perspective-shifting, decentering, ToM
• feedback integration
• scaffolding and transfer
• integration into learning and identity, generalization

Efklides’ MASRL model and IMR for illness self-management have similarities and overlap on goal-directed regulation, monitoring, control, motivation, affect and feedback. MASRL also contributes bottom-up regulation on tasks and metacognitive experiences as feelings. In addition to domain specifics, IMR adds coping with symptoms, relapse prevention, stress vulnerability, medication management and social support. If hallucinations are symptoms, managing uncertainty, monitoring and calibration can have higher relevance.

Disorders in clinical settings are likely to produce stronger affective responses during metacognitive training, due to resulting symptoms and constraints. Indeed, MT-DI may target more errors from System-1. Thus, affect regulation is more important. During education, some possible emotions are frustration, anxiety, failure and satisfaction whereas clinically, shame, threat, stigma, fear, loss of agency and demoralization are also possible.

**Figure 1:**
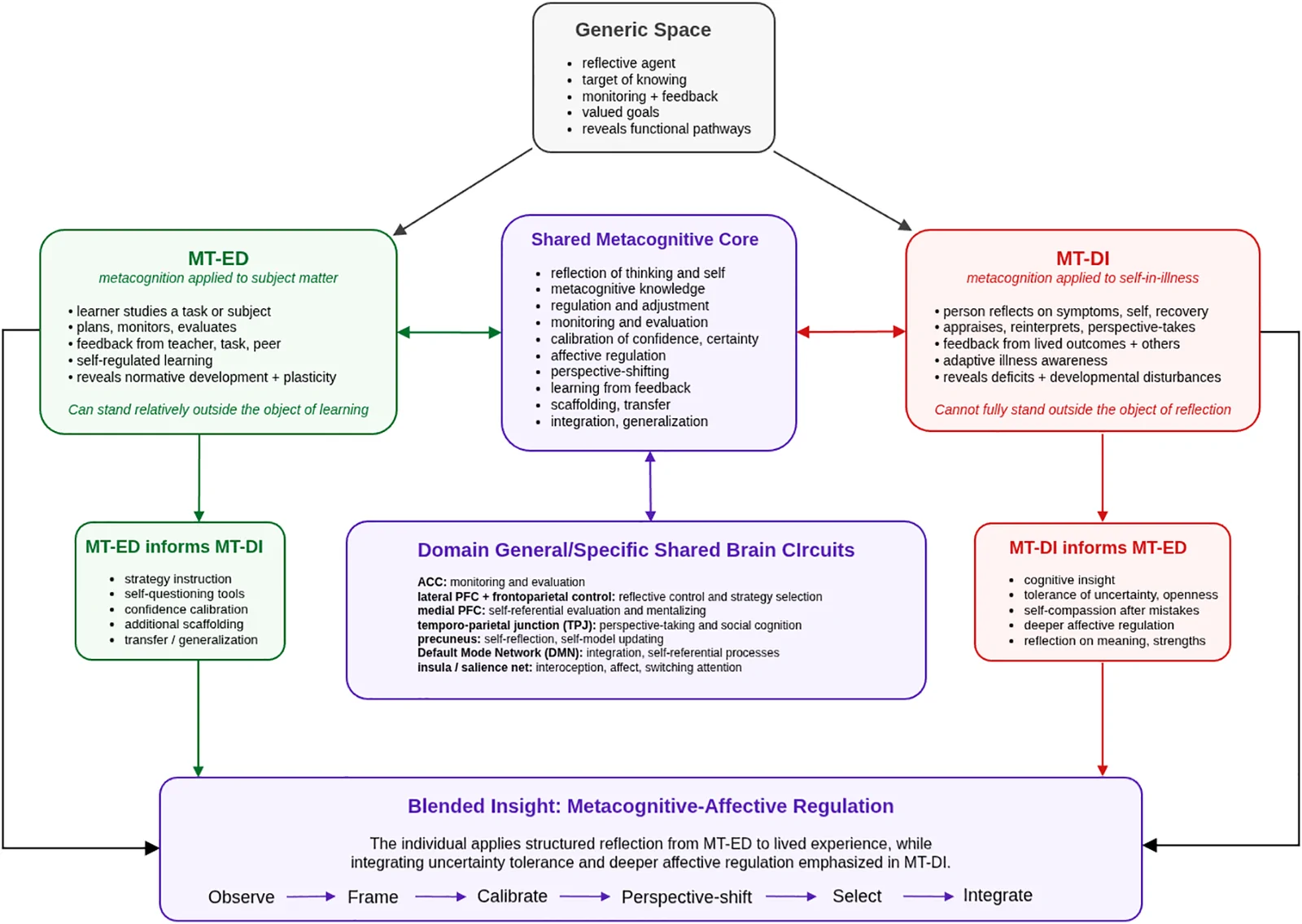
Conceptual blend of metacognitive training in education (MT-ED) and Metacognitive training for disease insight (MT-DI), showing how they overlap and how these approaches can inform each other.

MT-ED can inform MT-DI with enhancements on strategy instruction, self-questioning tools, reflective dialogs, scaffolding and generalization. MT-DI can inform MT-ED with additional aspects such as cognitive insight for addressing overconfidence, belief flexibility and self-certainty, self-compassion, openness, reflections on meaning, and tolerance for uncertainty, which is significant in psychosis. **Figure 1** shows a conceptual blend of MT-ED and MT-DI, encompassing what is shared, what is not and how these might be blended together.

By investigating the neural correlates and the educational traditions of metacognition, it may be possible to understand to what extent they share a common neural basis, as well as how constructs studied in the two disciplines are related (56). MT-ED can help identify neural correlates while MT-DI can indicate functional deficits that may show alterations to those correlates. While neurodevelopmental trajectories are considered differently in these contexts, both entail awareness of thought, goal-directed monitoring, managing uncertainty and learning from feedback.

## 6 Discussion

**Figure 2:**
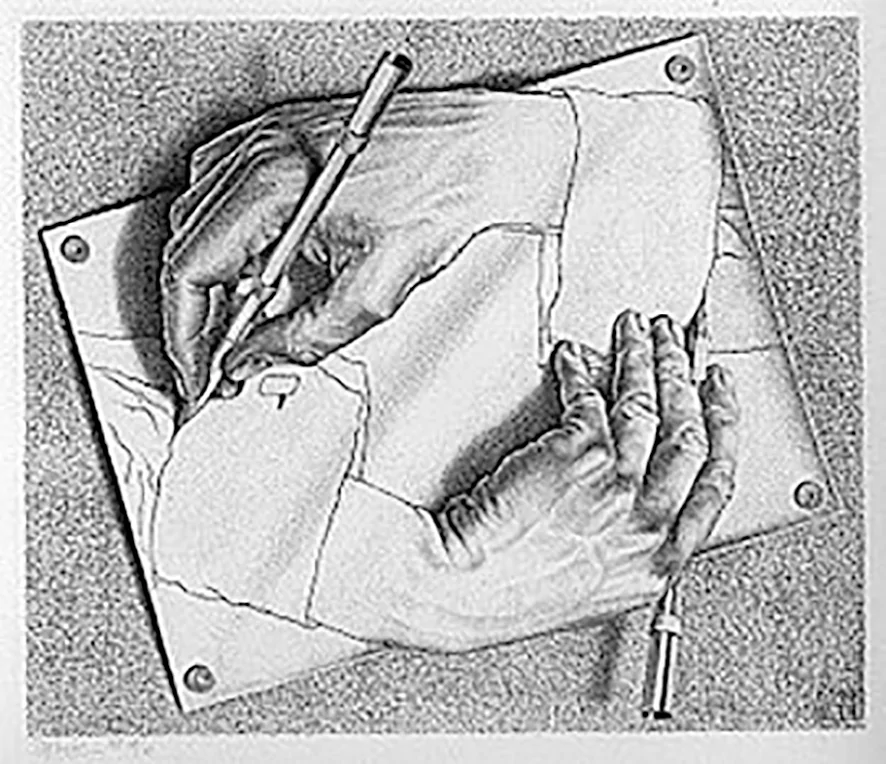
Lithograph "Drawing hands" from M. C. Escher from the “The Magic of M. C. Escher (1948).” Copyright was obtained for M.C. Escher’s “Drawing Hands” © 2026, from the “The M.C. Escher Company-The Netherlands. All rights reserved. www.mcescher.com PERMISSION NO: B-26/113. This metaphorically illustrates two self-referential processes which can inform each other.

In this perspective article, we raised the question of how educational and disease related insight and metacognition can better inform each other (56). We pinpointed overlapping neural subnetworks in the distributed metacognitive circuits and propose that understanding the failures of metacognition that occur following brain damage and psychiatric disorders could further inform educational disciplines and domains (51). Metacognitive skills development and training can significantly improve social skills, problem-solving abilities and performance. Similarly, educational settings like PBL and SRL are social and collaborative in methodology. Better patient education can improve treatment outcomes and reduce compulsory care, thus pedagogical approaches which improve metacognitive skills should be adjusted and re-invented for patients (42). Mindfulness training may support metacognitive reflection and affective regulation (78) and has been found to improve disease insight in schizophrenia (79, 80). Learning analytics for metacognition are emerging for both MT-ED (81) and MT-DI (38), which, using machine learning methods, can enable optimized and more personalized approaches in both educational and clinical settings. Further, incorporating neuroimaging data in such models may provide information gain for both predicting metacognitive function and improving clinical outcomes. We claim that mutual knowledge transfer between these disciplines can better delineate both domain-specific and domain-general cognitive mechanisms. Insight promotion interventions should address the trajectory of insight changes while assessing the possible detrimental effects, to maximize the beneficial but minimize the negative effects of increased disease insight (28). One possible approach for better synthesis is to investigate the gap between the two disciplines and the dialog should likely inform both fields, leading to better understandings of the metacognitive brain (**Figure 2**).

## Data Availability

The original contributions presented in the study are included in the article/supplementary material. Further inquiries can be directed to the corresponding author.
